# Subgroup differences in psychosocial factors relating to coronary heart disease in the UK South Asian population^☆^

**DOI:** 10.1016/j.jpsychores.2010.03.015

**Published:** 2010-10

**Authors:** Emily D. Williams, James Y. Nazroo, Jaspal S. Kooner, Andrew Steptoe

**Affiliations:** Department of Epidemiology and Public Health, University College London, London, UK

**Keywords:** Coronary heart disease, Psychosocial risk factors, South Asian, Subgroup differences

## Abstract

**Objectives:**

To explore the differences in psychosocial risk factors related to coronary heart disease (CHD) between South Asian subgroups in the UK. South Asian people suffer significantly higher rates of CHD than other ethnic groups, but vulnerability varies between South Asian subgroups, in terms of both CHD rates and risk profiles. Psychosocial factors may contribute to the excess CHD propensity that is observed; however, subgroup heterogeneity in psychosocial disadvantage has not previously been systematically explored.

**Methods:**

With a cross-sectional design, 1065 healthy South Asian and 818 white men and women from West London, UK, completed psychosocial questionnaires. Psychosocial profiles were compared between South Asian religious groups and the white sample, using analyses of covariance and post hoc tests.

**Results:**

Of the South Asian sample, 50.5% was Sikh, 28.0% was Hindu, and 15.8% was Muslim. Muslim participants were more socioeconomically deprived and experienced higher levels of chronic stress, including financial strain, low social cohesion, and racial discrimination, compared with other South Asian religious groups. In terms of health behaviors, Muslim men smoked more than Sikhs and Hindus, and Muslims also reported lower alcohol consumption and were less physically active than other groups.

**Conclusion:**

This study found that Muslims were exposed to more psychosocial and behavioral adversity than Sikhs and Hindus, and highlights the importance of investigating subgroup heterogeneity in South Asian CHD risk.

## Introduction

Cardiovascular disease rates vary by ethnic group [1]. In particular, coronary heart disease (CHD) rates are significantly higher in South Asian people (people originating from the Indian subcontinent) in the UK compared with other ethnic groups. This excessive vulnerability is evident in South Asians in most countries worldwide and is particularly marked in the younger age groups [2]. Despite evidence demonstrating significant heterogeneity in CHD risk among different groups of South Asians (subgroup heterogeneity), few studies have explored subgroup variations [3,4].

Some biological risk factors appear to demonstrate a general South Asian vulnerability, with all South Asian subgroups showing a predisposition toward diabetes and central obesity compared with the general population [4,5]. South Asians suffer elevated rates of insulin resistance [6], metabolic syndrome [7], and reduced high-density lipoprotein cholesterol [8] compared with other ethnic groups, although subgroup variations have been observed [7]. However, known biological risk factors do not appear to explain the higher CHD mortality of South Asian people compared with white Europeans in the UK [9]. South Asian subgroups vary markedly in terms of religion, culture, and language. In addition, their CHD risk factor profiles and CHD rates do not present a uniform distribution, with Bangladeshi and Pakistani groups experiencing significantly higher CHD morbidity than observed in Indians [2,3]. South Asians also show mixed profiles of health behaviors; smoking rates are especially high (men only) and alcohol consumption and levels of physical activity particularly low among Pakistani and Bangladeshi populations compared with Indian groups [10–12]. Positions in the social hierarchy in Britain differ between the South Asian subgroups too, with people originally from Bangladesh and Pakistan exposed to significantly higher social deprivation in comparison with Indian people [3].

Psychological and social factors show robust links with ill health, in particular CHD [13], but have not been comprehensively examined in UK South Asian subgroups. Depression, for example, is strongly associated with an increased risk of CHD development and poorer prognosis [14]. Chronic stressors, such as work stress, also relate to elevated CHD risk [15], while protective social factors, for example, social support, provide a buffer against psychosocial adversity [16]. Some studies have examined the psychosocial contribution to CHD risk in UK South Asians and have shown significantly higher psychosocial adversity in South Asians than in whites; however, there has been limited analysis of subgroup variation [17–20]. Previous research has shown that Pakistani and Bangladeshi/Muslim people experience greater socioeconomic disadvantage compared with other South Asian subgroups [3,7,21]; however, subgroup variation in other aspects of psychosocial risk has not been as well explored.

This study aimed to assess whether psychosocial profiles relating to the excessive CHD risk observed in UK South Asians varied across subgroups. Previous studies examining subgroup heterogeneity have categorized South Asian subgroups according to country of birth [3,7,22] and less commonly by religious affiliation [21,23]. We have used religious stratification because religion may be a stronger determinant of cardiovascular-related psychosocial and behavioral risk than country of birth, influencing health behaviors such as smoking, alcohol consumption and physical activity [23–26], and psychosocial adversity [21,27]. Accordingly, we compared psychosocial and behavioral factors that have been associated with CHD risk in a community sample of Sikh, Muslim, and Hindu people living in West London. We have previously shown that, compared with white Europeans, UK South Asians living in West London experience greater psychosocial adversity, in terms of financial strain, residential crowding, family conflict, social deprivation, and discrimination, while at the same time having lower social support and greater depression and hostility [20]. These differences were largely independent of socioeconomic position (SEP) as defined by household income. In the present analysis, the primary focus is on South Asian subgroup differences, rather than on comparisons with white Europeans.

## Methods

The methodology for this study has been described in detail elsewhere [20]. In brief, participants for this study were recruited from the London Life Sciences Prospective Population (LOLIPOP) study, a large cardiovascular risk assessment program in West London. Men and women aged 35–75 years were randomly selected from the LOLIPOP database for an intensive cardiovascular screening substudy of the LOLIPOP study and then stratified by age and ethnicity/ancestry (South Asian and white European people only). Documented CHD and other life-limiting illnesses were the exclusion criteria. Hospital appointment letters for cardiovascular tests were sent out to volunteers, accompanied by a psychosocial questionnaire (response rate: 83%). The questionnaire was available in English and Punjabi. Where understanding of Punjabi or English was not proficient, participants were offered the opportunity to complete the questionnaire with the help of a relative or friend, or were helped by a bilingual researcher on arrival at the hospital. The study was approved by the Ealing Hospital Local Research Ethics Committee, and written consent was obtained.

A total of 1948 male and female participants provided data. The final sample comprised 1130 South Asians (776 men, 354 women) and 818 white Europeans (606 men, 212 women).

### Questionnaire measures

These measures have been described in more detail elsewhere [20]. Briefly, the psychosocial assessment was divided into measures of socioeconomic factors, chronic stressors, protective factors in the social environment, and psychological variables. The questionnaire was tested in a preliminary interview-based study and was found to be comprehensible and feasible [28].

#### Socioeconomic position

Household income was the primary measure of SEP, grouped into tertiles: ≤£20,000, £20,000–£35,000, ≥£35,000. Information also regarding educational achievement, categorized as above or below secondary school, and age of leaving full-time education was obtained. Material deprivation was measured using an 11-item scale of household consumables, which was designed to be sensitive to SEP in ethnic minorities [3]. An adaptation of the Townsend Material Deprivation Index [29] based on self-reports of car and home ownership, residential overcrowding, and unemployment was used to assess social deprivation [30]. Scores ranged from 0 to 2, with 2 indicating elevated deprivation.

#### Chronic stress

Residential crowding was defined as living in a home with more than one person per room, as used in the US Census and elsewhere [31]. An adaptation of the economic strain scale of Pearlin et al. [32], using eight items, indicated levels of financial strain. Higher scores reflected greater financial strain (Cronbach *α*=0.91), with scores scaled from 0 to 100. Five items, developed for neighborhood studies in Chicago, assessed social cohesion, a marker of social capital [33]. Responses ranged from *very unlikely* to *very likely*, with scores ranging from 0 to 100 (Cronbach *α*=0.86). The Issues Checklist scale was modified to measure parental–child family conflict [34], with higher scores reflecting higher family conflict (Cronbach *α*=0.85) and scores ranged from 0 to 50.

Work stress was measured using the job strain and effort/reward imbalance models, with items from the scales used in the Whitehall II study [35]. Components of these models were each assessed with four to nine items and scaled to range from 0 to 100. Job strain was calculated by dividing demands by decision latitude (control plus skill discretion), and effort–reward imbalance by dividing effort by reward. Cronbach's *α* scores ranged from 0.55 to 0.88.

Racial discrimination was assessed using two measures. Firstly, a question enquiring whether participants had experienced any racially motivated attack in the last 12 months was posed, in terms of verbal abuse, physical attack, vandalism, or destruction to property [36]. Secondly, participants completed the perceptions of discrimination scale [37]; this scale included six questions measuring exposure to ethnically motivated discrimination (e.g., treatment by the police) over the last 5 years. Total discrimination scores (0 to 12) were created (Cronbach *α*=0.57).

#### Social relationships

Five questions from the social support inventory developed for the Enhancing Recovery in CHD (ENRICHD) study measured quality of social support [38], with scores ranging from 0 to 25 (Cronbach *α*=0.93). Negative aspects of social support were assessed with two items derived from the MacArthur social support scales (Cronbach *α*=0.68) [39]. Scores ranged from 0 to 8. The Social Network Index was used to measure social networks [40]. More diverse social networks were represented by greater values, ranging from 0 to 12.

Four items from the Santa Clara Strength of Religious Faith scale assessed religiosity in both ethnic groups [41]. A four-point scale rated religious beliefs from *strongly agree* to *strongly disagree*, with total scores ranging 0 to 12 (Cronbach *α*=0.93).

#### Psychological factors

Depression in the week preceding interview was measured using the 20-item Center for Epidemiologic Studies of Depression Scale (CES-D) [42]. Total scores ranged between 0 and 60; higher scores reflected greater depression (Cronbach *α*=.91). Cronbach's *α* scores for the LOT-R were 0.75 for whites but only 0.59 for South Asians. The Cook–Medley Hostility Scale was also administered (Cronbach *α*=0.81) [43]. Total scores ranged between 0 and 26.

#### Health behaviors

Information concerning smoking status was obtained, presenting *current smoker*, *ex-smoker*, and *nonsmoker* options. Weekly alcohol consumption was measured with units of different types of alcohol (e.g., wine, spirits), and a total score was calculated. Two simple questions were posed to measure diet. One question recorded the frequency with which the participant ate fresh fruit and vegetables using an eight-point scale, and another assessed the consumption of high- and low-fat products [44]. Questions from the International Physical Activity Questionnaire [45] measured sedentary behavior and physical activity. Sedentary behavior was measured with two questions asking the participant how much time they spent either ‘watching television/videos and/or playing computer games’ on weekend days and week days. The amount of time spent performing moderate exercise and vigorous exercise daily was also reported. These were combined to produce a categorical variable showing the number of days that the recommended amount of daily exercise (i.e., at least 30 min) was performed (none, 1–4 days, and 5 or more days).

### Statistical analysis

The psychosocial and behavioral CHD risk factors were examined across the South Asian subgroups of Sikhs, Muslims, and Hindus using analyses of covariance, controlling for age and sex. Where there were significant main effects of religion (*P* values presented in text), comparisons between pairs of groups (Sikhs, Muslims, Hindus, and whites) were performed post hoc using Fisher's least significant difference tests. The white group was included in these post hoc tests to establish whether the psychosocial differences observed previously between whites and South Asians [20] were true ethnic group differences or whether subgroup variation played an important role. In separate analyses, total household income was included as a covariate to establish whether the subgroup differences observed reflected socioeconomic variations. Preliminary analyses (not presented here) included sex as an additional factor. Where sex differences were evident, these effects have been explored separately by sex. In the tables, values are presented as means and percentages with 95% confidence intervals. Superscript letters indicate significant group differences (*P*<.05). All analyses were performed using the statistics program SPSS 14.0.

## Results

Of the South Asian sample, 50.5% was Sikh, 15.8% was Muslim, 28.0% was Hindu, and 3.2% was Christian. The large majority (94.9%) had been born outside the UK and had lived in the UK for an average of 29.2±11.7 years. Over two-thirds of the Sikhs were born in India and 16% were born in East Africa. Of the Muslim subgroup, 58.1% were born in Pakistan, 16% in India, and 12% in East Africa. Just over half of the Hindu participants originated from India, 26% were born in East Africa, and 12% were born in Sri Lanka. Over two-thirds of the South Asians spoke Punjabi as their mother tongue. The majority (81.3%) were married/cohabiting with partners. South Asian Christians were not included in the detailed comparisons.

Table 1 shows the demographic and socioeconomic group differences. The average age of respondents was 57.3 years (S.D. 10.0). There were no subgroup differences in age among South Asian men (*P*=.11); however, Hindu women were significantly older than Sikh and Muslim women (*P*<.001). Muslims tended to live in larger households than Sikhs and Hindus (*P*=.017), and Sikhs were the most likely and Muslims the least likely to own their homes (*P*<.001). Muslims had more children than other groups (*P*<.001). Because employment and education varied by sex, these variables were explored separately. Rates of employment did not differ between men (*P*=.157), although a larger proportion of Muslim men were self-employed compared with Sikh and Hindu men (*P*=.011). Muslim women were significantly more likely to be unemployed than other South Asian groups (*P*<.001). Educational attainment and age of leaving full-time education did not differ between the male and female South Asian subgroups. Lower income levels and elevated individual deprivation showed lower SEP was greater in Muslims than in other religious groups (*P*<.001).

### Chronic stressors

Muslim homes were marginally more overcrowded than Sikh and Hindu homes (*P*=.123). There were no differences between the South Asian groups in working hours per week (*P*=.130), and ratings of work stress did not vary across South Asian subgroups (Table 2).

Financial strain was markedly greater in Muslims than in Sikhs and Hindus (*P*<.001) and higher in all South Asian subgroups than in whites (*P*<.001), even after controlling for SEP (total household income) (*P*<.001). The social cohesion in the respondents' neighborhoods was lowest among the Muslim participants, although there was only a significant difference in comparison with Sikhs (*P*=.013). Family conflict did not differ between South Asian religions (*P*=.327). Twelve percent of Muslims had directly experienced racism, compared with 9% of Sikhs and 7% of Hindus; however, this was not statistically significant (*P*=.208). Both Muslims and Sikhs reported greater levels of racial discrimination than Hindus (*P*=.034). These chronic stress differences were independent of SEP.

### Social and psychological factors

No variations were observed in the quality of social support across the South Asian religious groups (*P*=.570). Negative support (e.g., perceptions of criticism from family) was higher in Sikhs compared with Muslims (*P*=.047), although this was no longer statistically significant after taking into account subgroup differences in SEP (*P*=.073). Sikhs had significantly larger social networks than Muslims and Hindus (*P*=.024), independently of socioeconomic factors. Muslim men and women reported stronger religious beliefs than Hindus (*P*=.038) (Table 3).

Levels of depression and hostility did not vary between the three South Asian religious groups.

### Health behaviors

Smoking rates were substantially higher among Muslim and Hindu men compared with Sikhs (*P*<.001). There was significant variation in alcohol consumption between the subgroups, with Muslims reporting the lowest levels, then Hindus, and then Sikhs (*P*<.001). Smoking and alcohol intake did not vary between South Asian females (*P*=.302 and *P*=.191, respectively). South Asian subgroups did not differ in their intake of fruit and vegetables (*P*=.106) or in their consumption of full-fat products (*P*=.164), while Muslims reported higher intake of reduced-fat products (*P*=.001). Muslims were significantly more sedentary than Sikhs and Hindus (*P*=.006), and were less likely to perform moderate or vigorous physical activity over the week (*P*=.007) (Table 4).

## Discussion

There is a limited amount of previous research that has examined socioeconomic and psychosocial risk factors across South Asian religious subgroups. Data from the Fourth National Survey of Ethnic Minorities demonstrated elevated socioeconomic adversity in Muslim groups compared with Sikh and Hindu people in the UK [22]. Subgroup differences have also been observed in levels of psychological distress between South Asian religious groups [46,47], with distress shown to be higher among Muslim women compared with women of other religious groups. Compared with this earlier research, the present study assessed a comprehensive range of psychosocial CHD risk factors in a community-based sample of three South Asian religious groups, employing a larger sample size than in previous community studies [46,47].

Our earlier analysis of this dataset showed that South Asian people suffer significant psychosocial adversity in comparison with UK whites [20]. The present analyses examined whether subgroup heterogeneity was important in understanding psychosocial adversity among South Asians in the UK. Previous research suggests that differences between South Asian subgroups may be crucial in understanding their true CHD risk [3], yet these differences have been largely overlooked in studies of CHD risk factors in South Asians. Previous studies have tended to focus on one particular subgroup [19] or use samples from specific occupational settings where the South Asian employees have tended to belong to one subgroup [17].

South Asian subgroups differ in terms of their CHD rates and risk factors [2,3,10]. The analyses in this study used religious affiliation to differentiate subgroups. This form of categorization was chosen in response to the literature detailing differences in health behaviors [23–26] and in socioeconomic and discrimination exposure between religious groups [22,27,48]. The findings indicate that profiles of psychosocial adversity vary across South Asian religious groups, and this may signify differing levels of psychosocial CHD risk. The Muslim people in this sample were the most psychosocially disadvantaged, followed by Hindus, and with Sikhs reporting the most favorable profiles of the South Asian subgroups. These findings are particularly interesting because the sample was taken from one geographical catchment area, suggesting that the environmental context did not vary a great deal between subgroups. Therefore any subgroup variations are the consequence of individual differences in cultural, socioeconomic, and psychosocial experience.

As shown in previous research [3,7,21,48], Muslim people were more likely to report poor socioeconomic circumstances, in terms of home ownership, income, and social deprivation. Chronic stressors were also higher among Muslims compared with Sikhs and Hindus, in the form of financial strain, overcrowding, and low levels of social cohesion. The South Asian population, in general, still remains the subject of racial discrimination in the UK [36]; however, the rise of ‘Islamophobia’ has led to an intensifying effect against British Muslims, particularly in the current political climate [27,49,50]. Racism has been investigated as a risk factor for ill health [36] and has been associated with subclinical atherosclerosis [51,52] and increased risk of myocardial infarction [36]. Social cohesion, a factor that has been associated with total mortality [53], has not been investigated in South Asian groups before. It does, however, correlate with socioeconomic status [33] and therefore is congruent with our findings and previous work [21] that Muslims are more disadvantaged than other South Asian subgroups. Interestingly, the subgroup heterogeneity in chronic stress risk factors was largely independent of SEP as defined by household income, indicating that the variations observed were the result of factors beyond socioeconomic differences.

No consistent differences in work stress defined either by the demand–control model of job strain or the effort–reward imbalance model were observed between South Asian subgroups. Work stress is a strong predictor of future CHD mortality and morbidity [16], and biological and behavioral mediators have been identified [54,55]. Identifying the reasons for the lack of differences would require more detailed examination of the occupational profile of the three groups. Nor were there differences in family conflict, although South Asians in general reported more conflict than white Europeans. Living in multigenerational homes, as is common in South Asian communities [47], helps to maintain traditional values. However, it can also be associated with considerable stress [56] and may explain the elevated family conflict among South Asians in this sample.

By contrast with other factors, Muslims were not substantially disadvantaged in relation to the other South Asian subgroups in the measures of social relationships; however, the patterns did not show enhanced protective social resources which might compensate for their elevated stress exposure. Emotional support is thought to protect against or buffer the effects of stress on health [57] and did not differ between the South Asian religious groups. This study, however, did find that Muslim participants reported stronger religious beliefs in comparison with the other South Asian religions, which supports previous work [58,59]. Some research suggests that religiosity provides protection against ill health [60,61], although the relationship between religious faith and cardiovascular disease is contentious.

The heightened exposure to psychosocial adversity observed in this Muslim population may contribute to the increased rates of CHD that have been recorded among UK Muslim people [62]. In addition, the high rates of smoking in Muslim men are likely to increase their CHD risk further compared with Hindus and Sikhs. These results support previous findings, showing distinctive patterns of smoking and alcohol use between South Asian subgroups [10,26]. The measures of physical activity mirrored earlier work, demonstrating lower levels of physical activity in South Asian people compared with whites and especially low levels in Muslim/Pakistani groups [63–65]. These behavioral patterns reflect the elevated risk of CHD observed among UK Muslim people [62].

The Sikh population in this study showed some signs of psychosocial advantage compared with the other subgroups. For example, Sikh men and women reported greater home and car ownership, higher household incomes, and lower levels of deprivation. Sikhs were also less likely to experience chronic stressors, reporting lower financial strain and higher social cohesion, although their ratings of racial discrimination were as high as those of Muslims. Interestingly, Sikhs experienced greater negative support from close family and friends; high levels of negative interactions have been shown to relate to detrimental physiological responses [66,67]. Conversely, social network diversity was significantly greater in Sikhs compared with all other groups.

Sikhs were at lower cardiovascular risk by virtue of smoking less, yet reported higher levels of alcohol consumption than the other religious subgroups. Alcohol intake was still, however, relatively low and may fall into the protective range of consumption [68]. Since there have been no previous investigations of the psychosocial experience of South Asian subgroups on this scale, this appears to be the first study to show an advantage for Sikhs compared with other South Asian religious groups. To date, there has also been limited research on CHD rates among Sikh people in the UK, so it is not clear whether the psychosocial and behavioral advantages observed in this study translate to reduced CHD risk among the UK Sikh population. Previous work has, however, observed lower risk of CHD among Indian groups in the UK compared with other South Asian subgroups [62], and therefore since over two-thirds of the Sikh sample in this study was born in India, our findings support this.

In general, the Hindu people in this study closely paralleled Sikhs in their psychosocial profiles. Hindus had fewer children than other South Asian groups and had high levels of employment and home ownership. They reported the lowest racism and discrimination of all the South Asian groups and lower levels of religious beliefs. Despite being slightly older on average than the Sikhs and Muslims in this study, these findings indicate the possibility of greater westernization among the Hindu population. There is mixed evidence about the role of westernization in CHD risk; some studies show poorer health is aligned with greater westernization [69], while other work implicates the health benefits of acculturation [70].

In the primary comparison between South Asian and white European groups, we showed marked differences in psychological characteristics, with higher hostility and depression scores [20]. There was little subgroup variation in these psychological characteristics, with all three religious subgroups displaying elevated levels in comparison with UK whites. This is in contrast to previous work that has presented subgroup variation in depression [46,71]. The reasons for the discrepancies are not clear, but may relate to sample selection or measurement tools. Depression and hostility have been linked with increased CHD rates and CHD risk factors [14,72].

The strengths of this study were the large sample of South Asian participants from each subgroup, the use of standardized measures, and recruitment from the community. However, some limitations should be considered. Response rates may have been affected by health selection and socioeconomic biases which could have influenced the representative nature of the sample. Questionnaire completion meant the data were open to self-report bias. Most of the psychosocial scales used in health research have not been comprehensively validated in populations other than white, western groups. Statistical validation of the scales was performed in this bicultural sample (unpublished data), but it is still possible that interpretation bias was present and that people responded to some questions in different ways [73]. Some of the scales included may also have been influenced by the participant's mood at the time of completion; however, adjustment for concurrent negative affect was performed in earlier analyses and did not affect the group differences observed.

The results from this study demonstrate that, despite living in similar local environments, there is important heterogeneity in psychosocial and behavioral experiences between South Asian religious subgroups. Although the strongest differences are between whites and South Asians, the inter-religious variation observed highlights the relevance of studying subgroup heterogeneity and indicates that a clearer understanding and assessment of the concepts of ethnicity and religion will increase our awareness of health differences between groups. This work further endorses the importance of taking psychosocial profiles into account when assessing South Asian CHD risk and suggests that, where possible, subgroups should be examined separately. CHD risk is not uniform across South Asian subgroups [3] and therefore subgroup heterogeneity in psychosocial profiles should be examined in detail.

## Figures and Tables

**Table 1:** Demographic and socioeconomic information

	Age- and sex adjusted			
	Sikhs (*n*=571)	Muslims (*n*=179)	Hindus (*n*=315)	Whites (*n*=818)
Age (in years)	55.0 (54.2–55.9)^a^	55.2 (53.7–56.7)^a^	57.6 (56.5–58.7)^b^	57.2 (.56.5–57.9)^b^
Marital status—Married	90.1% (86.9–93.3)^a^	90.1% (84.4–95.9)^a,b^	84.1% (79.7–88.4)^b^	67.9% (65.2–70.6)^c^
No. of children	2.63 (2.5–2.7)^a^	3.03 (2.9–3.2)^b^	2.33 (2.2–2.5)^c^	1.94 (1.8–2.0)^d^
No. of people in household	4.31 (4.2–4.4)^a^	4.32 (4.1–4.6)^a^	4.01 (3.8–4.2)^b^	2.77 (2.7–2.9)^c^
Home ownership—Owner	86.9% (83.8–90.1)^a^	69.9% (64.2–75.6)^b^	80.9% (76.6–85.2)^c^	81.4% (78.8–84.0)^c^
Car ownership—Owner	81.9% (78.7–85.0)^a^	73.6% (67.9–79.3)^b^	73.5% (69.2–77.8)^b^	83.7% (81.0–86.3)^a^
Employment—Working	61.6% (58.3–65.0)^a^	49.2% (43.2–55.1)^b^	63.6% (59.1–68.1)^a,c^	67.1% (64.3–69.8)^c^
Self-employed	14.9% (11.0–18.8)^a^	28.6% (20.9–36.3)^b^	14.1% (8.6–19.5)^a^	20.5% (17.3–23.7)^b^
Age of leaving full-time education	19.3 (18.9–19.7)^a^	19.6 (18.9–20.3)^a^	19.2 (18.7–19.7)^a^	17.8 (17.5–18.1)^b^
Individual deprivation (an adaptation of Townsend deprivation index)	.62 (.57–.68)^a^	.90 (.79–1.0)^b^	.71 (.63–.79)^a^	.39 (.34–.44)^c^

Same superscript letters indicate no significant difference between religious subgroups. Different superscript letters indicate significant difference between subgroups, *P*<.05.

**Table 2:** Chronic stressors

	Age- and sex adjusted			
	Sikhs (*n*=571)	Muslims (*n*=179)	Hindus (*n*=315)	Whites (*n*=818)
Overcrowding	33.0% (29.9–36.1)^a^	40.1% (34.5–45.6)^b^	30.8% (26.7–35.0)^a^	3.9% (1.3–6.5)^c^
Work hours per week	40.3 (39.2–41.5)^a^	37.9 (35.5–40.2)^a^	40.6 (39.0–42.2)^a^	38.9 (37.9–40.0)^a^
Work stress				
Job strain	1.13 (.92–1.3)^a^	1.13 (.69–1.57)^a^	1.46 (1.2–1.8)^a^	1.17 (1.0–1.3)^a^
Effort–reward imbalance	1.64 (1.5–1.8)^a,b^	1.62 (1.3–2.0)^a,b^	1.82 (1.6–2.1)^a^	1.52 (1.4–1.7)^b^
Work support	40.7 (38.4–43.0)^a,b^	44.9 (40.1–49.8)^a,b^	43.1 (39.7–46.4)^a^	50.0 (47.9–52.0)^b^
Financial strain	3.54 (3.2–3.9)^a^	5.06 (4.5–5.6)^b^	3.90 (3.5–4.3)^a^	3.01 (2.7–3.3)^c^
Social cohesion	60.8 (59.2–62.4)^a,b^	56.2 (53.4–59.1)^c^	58.4 (56.2–60.5)^a,c^	62.2 (60.8–63.5)^b^
Family conflict	12.28 (11.4–13.1)^a^	10.83 (9.2–12.4)^a,b^	11.5 (10.3–12.7)^a^	9.17 (8.3–10.1)^b^
Experience of racism	9.3% (7.1–11.4)^a,b^	11.7% (7.9–15.5)^a^	6.7% (3.8–9.6)^b,c^	
Discrimination	38.1% (34.5–41.7)^a^	39.2% (32.6–45.8)^a^	28.6% (23.7–33.4)^b^	

Same superscript letters indicate no significant difference between religious subgroups. Different superscript letters indicate significant difference between subgroups, *P*<.05.

**Table 3:** Social and psychological characteristics

	Age- and sex adjusted			
	Sikhs (*n*=571)	Muslims (*n*=179)	Hindus (*n*=315)	Whites (*n*=818)
Social support	19.2 (18.8–19.6)^a^	19.4 (18.7–20.1)^a^	19.0 (18.5–19.5)^a^	20.2 (19.9–20.6)^b^
Negative support	3.09 (2.9–3.2)^a^	2.71 (2.4–3.0)^a,b^	2.89 (2.7–3.1)^a^	2.54 (2.4–2.7)^b^
Social network	5.56 (5.4–5.7)^a^	5.11 (4.8–5.4)^b^	5.25 (5.0–5.5)^b^	5.02 (4.9–5.1)^b^
Strength of religious beliefs	7.52 (7.2–7.8) ^ab^	8.01 (7.5–8.5)^a^	7.14 (6.8–7.5)^a,b^	5.58 (5.3–5.8)^b^
Depression	15.2 (14.4–16.1)^a^	15.2 (13.7–16.7)^a^	14.7 (13.6–15.8)^a^	12.0 (11.3–12.7)^b^
Hostility	13.9 (13.5–14.4)^a^	13.3 (12.6–14.1)^a^	13.2 (12.7–13.8)^a^	11.4 (11.1–11.8)^b^

Same superscript letters indicate no significant difference between religious subgroups. Different superscript letters indicate significant difference between subgroups, *P*<.05.

**Table 4:** Health behavior characteristics

	Age- and sex adjusted			
	Sikhs (*n*=571)	Muslims (*n*=179)	Hindus (*n*=315)	Whites (*n*=818)
Smoking				
Ever smoked	8.7% (5.2–12.1)^a^	32.1% (26.0–38.3)^b^	26.5% (21.8–31.1)^b^	55.4% (52.6–58.3)^c^
Currently smoke	4.1% (1.4–6.8)^a^	18.1% (14.0–23.6)^b^	10.6% (7.0–14.3)^c^	18.8% (16.5–21.0)^b^
Alcohol intake (weekly)	6.82 (5.8–7.9)^a^	1.21 (–.68–3.1)^b^	4.91 (3.5–6.3)^c^	13.8 (12.9–14.7)^d^
Fruit/vegetable intake—more than 1/day	26.7% (22.8–30.6)^a^	19.6% (12.6–26.6)^a^	28.1% (22.8–33.3)^a^	42.0% (38.7–45.3)^b^
Low fat products—Always	58.6% (54.6–62.3)^a^	73.3% (66.1–80.5)^b^	61.7% (56.3–67.1)^a^	52.2% (48.8–55.5)^c^
Full-fat products—Always	16.1% (13.3–18.8)^a^	12.1% (7.2–17.0)^a,b^	18.0% (14.3–21.6)^a^	8.7% (6.4–11.0)^b^
Sedentary behavior—more than 3 h/day	44.2% (40.0–48.4)^a^	54.9% (47.3–62.5)^b^	43.1% (37.3–48.8)^a^	47.5% (44.0–51.1)^a,b^
Physical activity— Some	74.2% (70.5–77.8)^a^	63.4% (56.9–70.0)^b^	77.3% (72.4–82.2)^a,c^	79.4% (76.3–82.4)^c^

Same superscript letters indicate no significant difference between religious subgroups. Different superscript letters indicate significant difference between subgroups, *P*<.05.
